# ACBD3 Bioinformatic Analysis and Protein Expression in Breast Cancer Cells

**DOI:** 10.3390/ijms23168881

**Published:** 2022-08-10

**Authors:** Jack Houghton-Gisby, Rachel Kerslake, Emmanouil Karteris, Kefah Mokbel, Amanda J. Harvey

**Affiliations:** 1 Centre for Genome Engineering and Maintenance, Institute for Health Medicine and Environments, Brunel University London, Uxbridge UB8 3PH, UK; 2 Princess Grace Hospital, The London Breast Institute, London, W1U 5NY, UK

**Keywords:** ACBD3, breast cancer, chromosome 1q, patient outcomes

## Abstract

*ACBD3* overexpression has previously been found to correlate with worse prognosis for breast cancer patients and, as an incredibly diverse protein in both function and cellular localisation, ACBD3 may have a larger role in breast cancer than previously thought. This study further investigated ACBD3′s role in breast cancer. Bioinformatic databases were queried to characterise *ACBD3* expression and mutation in breast cancer and to investigate how overexpression affects breast cancer patient outcomes. Immunohistochemistry was carried out to examine ACBD3 location within cells and tissue structures. *ACBD3* was more highly expressed in breast cancer than in any other cancer or matched normal tissue, and expression over the median level resulted in reduced relapse-free, overall, and distant metastasis-free survival for breast cancer patients as a whole, with some differences observed between subtypes. IHC analysis found that ACBD3 levels varied based on hormone receptor status, indicating that ACBD3 could be a candidate biomarker for poor patient prognosis in breast cancer and may possibly be a biomarker for ER signal reprogramming of precancerous breast tissue.

## 1. Introduction

*ACBD3* (acyl-CoA binding domain containing protein 3) encodes a ubiquitously expressed protein of the same name that has an unusually large number of cellular roles. ACBD3 is essential for embryonic development, and *ACBD3−/−* knockout is reported to be embryonic-lethal in mice between 8.5 and 10.5 days [1], whilst human K562 erythroleukemia cells arrest in G_1_ when ACBD3 is downregulated by siRNA [2]. ACBD3 is considered a Golgi resident protein and contains a Golgi dynamics (GOLD) domain which localizes ACBD3 to this organelle where it interacts with Golgin subfamily B member 1 (Giantin) [3], GOLGIN-160 [4,5,6], Golgin-45 [7], protein phosphatase 1L [8], TBC1 domain family member 22A/B [7], and the most commonly ACBD3-associated protein phosphatidylinositol 4-kinase beta (PI4Kβ) [9]. These interactions are crucial for Golgi stacking structure and function. Additionally, ACBD3 interacts with TUG (tether containing UBX domain for GLUT4), a protein that tethers GLUT4 storage vesicles at the Golgi membrane and releases them in response to insulin signaling, allowing glucose import into the cell [4,10]. Of note, both GLUT4 and the insulin receptor are being investigated as targets for breast cancer therapy [11,12]. ACBD3 overexpression could feasibly increase capacity for GLUT4 storage and maximize the Warburg effect facilitated by GLUT4.

ACBD3 localizes to other membranes, including the outer mitochondrial membrane (OMM), where it interacts with the transmembrane TSPO translocator protein and forms complexes with protein kinase A (PKA) through the R1α regulatory subunit. The TSPO-ACBD3-PKA holoenzyme complex phosphorylates and activates the steroidogenic acute regulatory protein (StAR), which catalyzes import of cholesterol across the OMM and is the rate limiting step in steroid synthesis [13,14]. The PKA regulatory subunit, PKAR1α, has previously been reported to be upregulated in cancer cell lines, and the implications of dysregulating steroid synthesis in breast cancer are plain [15,16].

During mitosis, the Golgi fragments and ACBD3 are released into the cytosol; in embryonic development, cytosolic ACBD3 inhibits NOTCH signaling in dividing neuronal precursor cells to maintain a pool of these stem cells so that the brain may develop fully [17]. ACBD3 achieves this by binding the NUMB protein, which increases its affinity for NOTCH; the NUMB–NOTCH interaction then inhibits NOTCH and prevents signaling. *NOTCH1*, *NOTCH3*, and *JAG1* expression are associated with poor survival in breast cancer patients [18], and NOTCH overexpression transformed MCF10A breast cells whilst overexpression of NUMB reversed this transformation [19]. ACBD3 reduces NOTCH signaling in conjunction with NUMB, but NUMB and NUMB-L are downregulated in breast cancers [20] and NUMB-deficient breast cancer cells form increased cancer stem cell (CSC) pools [21], suggesting that ACBD3 may promote CSCs independently of NUMB.

Increased *ACBD3* expression has previously been found to correlate with poor breast cancer patient prognosis and is overexpressed in commercial breast cancer cell lines [22]. *ACBD3* overexpression in T47D and BT549 breast cell lines caused increased, bulkier mammosphere formation in suspension cultures, whilst silencing of *ACBD3* with siRNA reduced the size and number of mammospheres [22]. Similarly, flow cytometry data suggested that *ACBD3* overexpression increased CSC populations [22]. Huang and colleagues concluded that ACBD3 activated the Wnt/β-catenin signaling pathway and that this was causative of CSC side population maintenance and malignant mammosphere formation.

The mechanism by which ACBD3 promotes CSCs and worsens patient outcomes is not understood and may be much broader than activation of the Wnt/β-catenin signaling pathway. The role of ACBD3 is highly contextual depending on partners and cellular location, and ACBD3 has a lack of known redundancies for many of its functions [1,23]. ACBD3 has many interactors, some of which are implicated in breast cancer in their own right [11,19,20,21,24,25,26,27,28]. Many roles of ACBD3 could arguably promote the hallmarks of cancer, including dysregulating cellular energetics, sustaining proliferative signaling, replicative immortality, and tumor-promoting inflammation [4,17,29,30,31,32,33]; the location of *ACBD3* on arm q of chromosome 1 may also be important, as 1q amplification is common in breast cancer, and the *ACBD3* locus (1q42.13) is within a the largest region of gain [34].

As direct evidence for the mechanisms that correlate ACBD3 expression and cancer prognosis are very limited, we have used in silico tools to provide a better insight of ACBD3 expression, regulation, and mutational landscape in healthy and cancerous breast tissue. In addition, databases were queried to investigate the link between ACBD3 expression and survival, relapse and metastatic outcomes for patients divided by receptor status, breast cancer subtype, and response to chemotherapeutic agents. Finally, ACBD3 expression in normal, adjacent, and cancerous tissue was assessed to examine possible relationships between breast cancer subtypes and patient characteristics.

## 2. Results

### 2.1. ACBD3 Expression in Tumours and Normal Tissue

**Figure 1:**
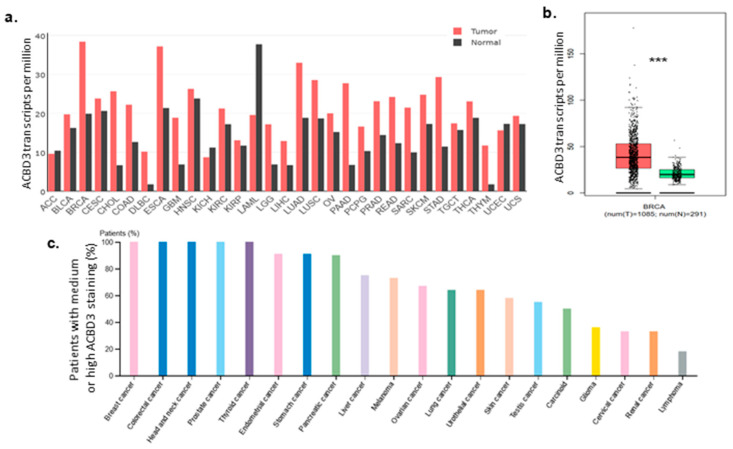
ACBD3 mRNA and protein expression in cancers. (**a**) Median *ACBD3* mRNA expression in transcripts per million in different tumours and matched normal tissue [35]. (**b**) *ACBD3* mRNA in breast tumour samples (red) and paired normal breast tissue (green) (*** *p* = < 0.001, Log_2_FC cutoff = 0.75). *ACBD3* expression was 93.06% higher in cancerous breast tissue and samples also had a larger range of expression than normal tissue. (**c**) Protein levels of ACBD3 in cancers, measured by medium or high antibody staining as a percentage of total patient samples.

The gene expression profiling interactive analysis tool (GEPIA) was queried for *ACBD3* transcription levels in different tissues, and most normal tissues were found to have lower expression of *ACBD3* than their paired tumor samples. *ACBD3* mRNA was expressed at 19.88 transcripts per million (TPM) in normal breast tissue and was almost two-fold greater in paired invasive breast carcinoma at 38.38 TPM. Transcription was higher in breast cancer than in any other cancer or paired healthy tissue (Figure 1a) [35]. Breast cancer *ACBD3* expression had a larger interquartile range, smaller minimum value, higher maximum value, and more numerous and distant outliers beyond the maximum range and were significantly higher than in matched healthy tissue (Figure 1b). Conversely, *ACBD3* mRNA expression in acute myeloid leukaemia was lower than its paired normal tissue (37.71 vs. 19.56 TPM), a very close inverse to the breast *ACBD3* expression profile, potentially suggesting differences in role or context of ACBD3 function between solid and haematopoietic tumors. Three other tumor types, adrenocortical carcinoma, kidney chromophobe, and uterine corpus endometrial carcinoma, showed downregulation of *ACBD3* mRNA expression compared to matched normal tissue.

The human protein atlas was queried to investigate whether high levels of ACBD3 protein were also found in cancers. Whilst a direct correlation was not expected, it was reassuring that tissues with higher mRNA expression levels such as prostate and colon cancer, as well as head and neck carcinoma samples, also had higher protein levels. Breast cancer had one of the highest levels of ACBD3 protein expression by the methodology used (11 out of 11 patient samples had medium levels of ACBD3 staining) (Figure 1c).

### 2.2. ACBD3 Gene Amplification and Mutation in Cancer

**Figure 2:**
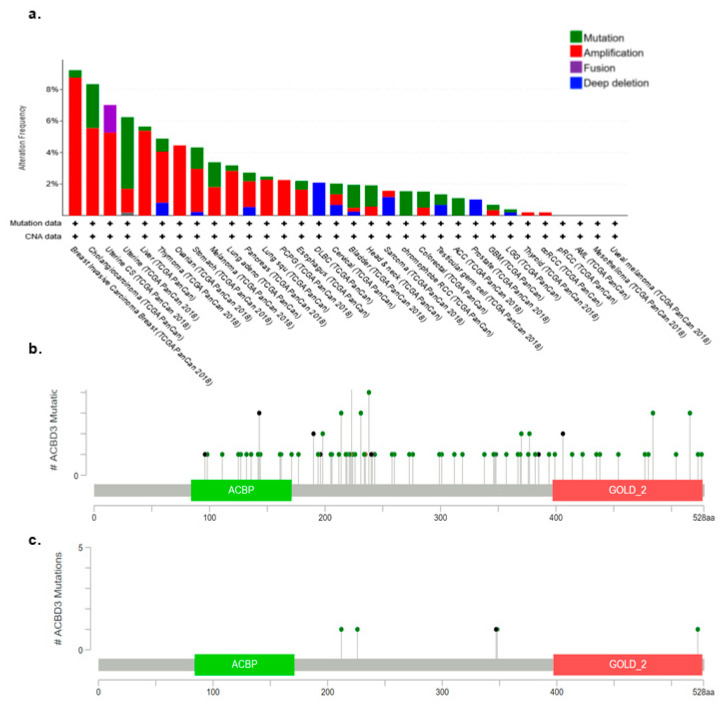
The frequency of *ACBD3* genetic alterations in cancers. (**a**) *ACBD3* is mutated infrequently in breast cancers but is amplified more in breast cancer than in any other cancer. *ACBD3* is most frequently mutated in adrenocortical carcinoma. (**b**) Position and frequency of mutations in ACBD3 that result in amino acid changes for all cancers and (**c**) breast cancers. The ACBP and GOLD domain are highlighted; ACBD3 somatic mutation frequency in breast cancers was 0.5%.

The cBioPortal for cancer genomics was used to examine the alteration frequency of ACBD3 in terms of mutation, amplification structural variants, deep deletions, and multiple alterations. [36,37]. Breast cancers were found to have the highest proportion of *ACBD3* gene amplifications, at 8.76%, relative to other cancers such as uterine and prostate (Figure 2), and mutation frequency was low across all the cancers examined. In breast cancer, *ACBD3* mutations occurred in 5 out of 1084 patients; these included E212Q, E226K, E348Q, and R523T mutation, as well as a E348Nfs*21 frame shift deletion (Figure 2c). *ACBD3* is located on chromosome 1 arm q, which is frequently amplified in breast cancers. The amplification rate of *ACBD3* in breast cancer was less than expected based on the *ACBD3* transcriptional upregulation observed with the GEPIA tool (Figure 1) and the commonality of chromosome 1q amplification in breast cancer [34].

Previous work by Buniello and colleagues used the Genome Wide Association Studies catalogue for phenotypic risk association [38] and found that five DNA variants upstream of *ACBD3* (three intergenic variants and two regulatory region variants) were associated with core binding factor acute myeloid leukaemia risk [39]. There was one other risk variant, but this was not linked to cancer but rather behaviour which is not unexpected given that several other ACBD family proteins are known to influence behaviour in animals [40,41,42]. The Genehancer database was also searched, corroborating that the same five *ACBD3* variants were associated with core binding factor acute myeloid leukaemia risk; additionally, *ACBD3* regulatory region variants were found that enhanced the red blood cell distribution width (a measure of red blood cell volume variation), and variants that are associated with plateletcrit (the percentage of blood volume occupied by platelets) [43]. Genehancer additionally found an *ACBD3* regulatory region variant that was associated with DNA methylation. As upstream DNA methylation can affect the transcription level of genes, mutation here could increase ACBD3 transcription without altering the gene itself.

### 2.3. Copy Number Variation and Promoter Methylation of ACBD3 in Breast Cancer

**Figure 3:**
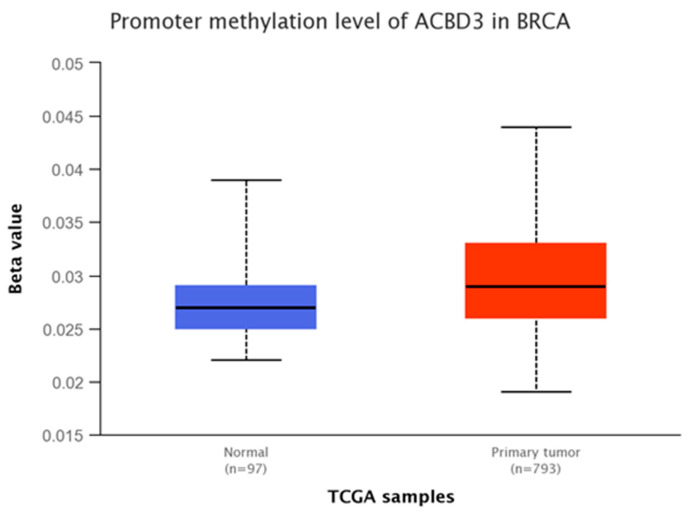
*ACBD3* promoter methylation in normal breast tissue (blue) and breast tumor tissue (red). A low beta value represents low methylation and therefore low inhibition of transcription. A beta value below 0.3 is considered hypomethylation. ACBD3 promoter methylation was very low in both normal and tumor breast tissue with no significant difference. Gene regulation by methylation cannot account for changes to *ACBD3* expression found in breast cancers.

The UALCAN resource was queried to determine the mechanisms underlying this upregulation. Methylation of the *ACBD3* promoter region was examined using TCGA and MET500 databases [44], as variations in methylation of gene promoters are linked with transcriptional regulation, typically repression [45,46,47,48,49,50]. In this study, tumor sample *ACBD3* promoter methylation was not found to be significantly different from paired normal tissue methylation (* *p* = 0.86), and methylation was observed to be very low in both cases (Figure 3). Based on these datasets, it is suggestive that the *ACBD3* reading frame is constitutively open and accessible in healthy and cancerous breast tissue and that methylation is not a major regulator of the *ACDB3* gene expression. *ACBD3* promoter methylation was similarly low in all other tissues examined (data not shown).

### 2.4. ACBD3 Transcription Factors in Breast Tissue

**Figure 4:**
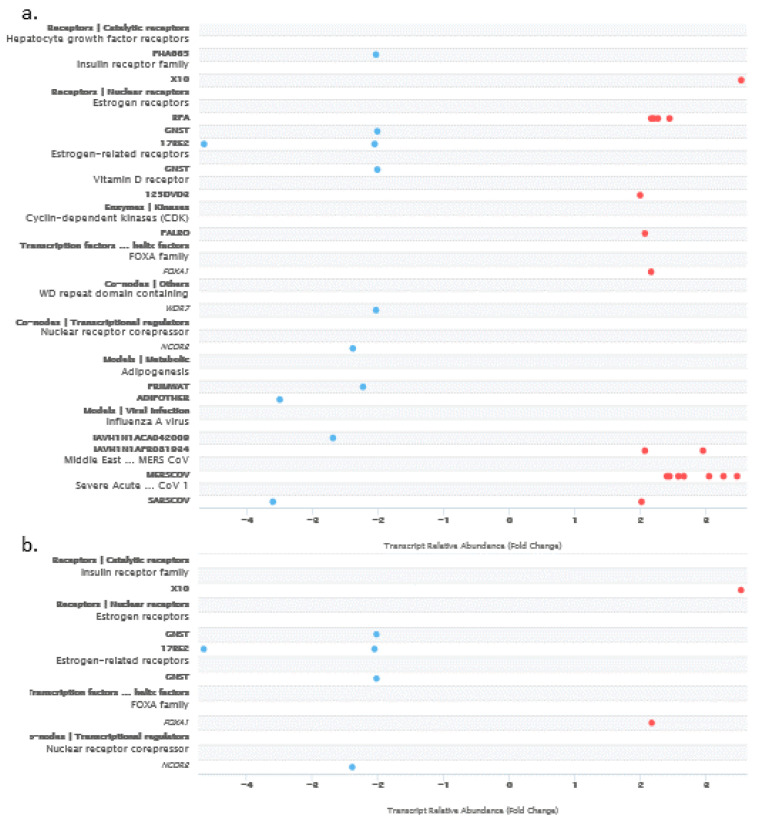
Receptor, enzyme, and transcription factor pathway nodes, small molecules, and viruses that regulate *ACBD3* transcription. Blue dots represent repression of *ACBD3* transcripts and red dots represent promotion of *ACBD3* transcripts; the cut-off was set at a transcription fold change of at least 2 and a confidence interval of 99.9% or greater. (**a**) Factors that affect *ACBD3* transcription across all normal tissues. (**b**) Factors that affect *ACBD3* transcription in normal breast tissue.

As both methylation and amplification of *ACBD3* were low in breast cancer samples compared to the level of *ACBD3* upregulation, the signaling pathways project (SPP) ChIPseq database was used to find *ACBD3* binding factors in normal tissue that may be important in breast cancer [51]. A large number of factors were discovered that bind within 10,000 bases of the *ACBD3* transcription start site across all tissues, and some of these stand out as having roles in breast cancers, including NOTCH1-NICD, CDK9, CTCF, and CEBPB [18,19,52,53,54,55,56,57,58,59,60,61,62]. The transcriptomics function of the SPP was used to find regulators of *ACBD3* expression that caused a fold change of two or more for all tissues (Figure 4a). Amongst those identified across tissue types were the oestrogen receptor (when stimulated with bisphenol A), the insulin receptor, the vitamin D receptor, FOXA1, and a number of viral transcription factors. In breast tissue specifically, the insulin receptor pathway related X10 ligand and the FOXA1 transcription factor were shown to increase *ACBD3* transcription by two-fold or more (Figure 4b).

### 2.5. ACBD3 mRNA Expression and Breast Cancer Patient Prognosis

Previously, high *ACBD3* tumor expression was correlated with poorer overall survival for breast cancer patients irrespective of clinical stage [22], but little is known about the impact of ACBD3 expression in the different breast cancer subtypes, or on either relapse-free or distant metastasis-free survival. The KMplotter breast cancer mRNA gene chip database was used to look at differences in survival, relapse, and distant metastasis in breast cancer patients based on whether mRNA levels of *ACBD3* were above or below the median expression level in their breast tumor [63].

**Figure 5:**
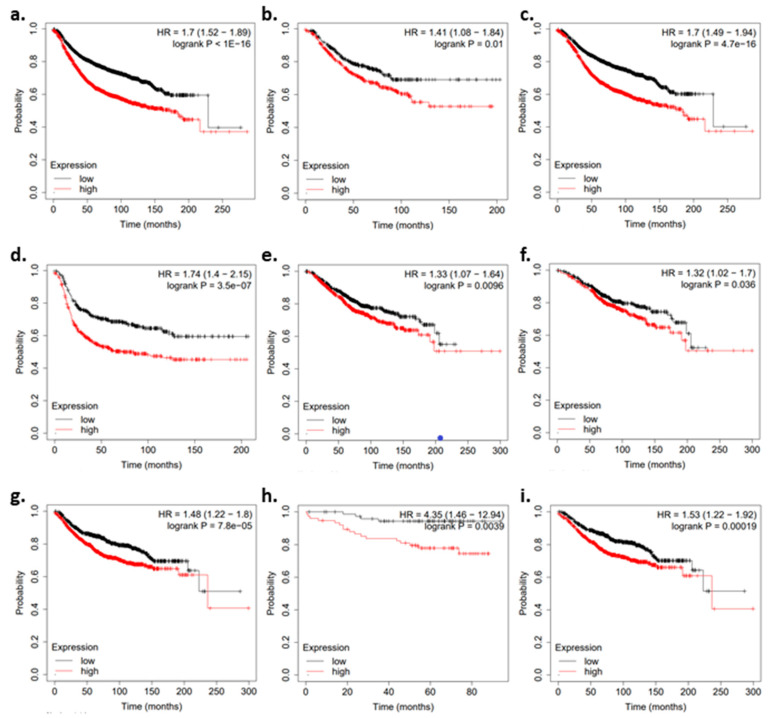
Kaplan–Meier plots for patient prognosis when divided by *ACBD3* mRNA expression. Black data points represent patients whose breast tumors had *ACBD3* mRNA expression below the median level. Red data points represent patients whose breast tumors had *ACBD3* mRNA expression above the median level. (**a**–**d**) Relapse-free survival when *ACBD3* is high or low for (**a**) breast cancer patient cohort overall, (**b**) HER2− breast cancer patients, (**c**) ER+ breast cancer patients, (**d**) ER−breast cancer patients. (**e**,**f**) Overall survival when tumor *ACBD3* is high or low for (**e**) the breast cancer patient cohort overall, (**f**) ER+ breast cancer patients. (**g**–**i**) Overall distant metastasis-free survival when tumor *ACBD3* was high or low for (**g**) the breast cancer patient cohort overall, (**h**) HER2− breast cancer patients, (**i**) ER+ breast cancer patients.

Higher *ACBD3* levels were associated with earlier relapse, more probable distant metastasis, and lower survival. When exploring subgroups and tumor subtypes, high *ACBD3* expression was associated with a higher probability of relapse and distant metastasis in patients. High tumor *ACBD3* was also associated with less overall survival in HER2- tumors (Figure 5).

Median relapse free survival (RFS) survival was 229 months when *ACBD3* was expressed below the median and 173 months when *ACBD3* expression was above the median (Figure 5a). RFS was significantly lower in HER2− patients with *ACBD3* expression above the median (43 months) compared to below the median (74 months) (Figure 5b). RFS was not significantly different when triple negative breast cancer patients were divided by median *ACBD3*, but both ER+ and ER− groups had less RFS when tumor *ACBD3* was expressed above the median (Figure 5c,d). RFS was also not significantly different when PR+ and PR- patients were divided by *ACBD3* expression.

The probability of overall survival (OS) was reduced in breast cancer patients with tumor *ACBD3* expression above the median level (Figure 5e). Upper quartile survival was 126 months for patients when *ACBD3* was below the median level and 82 months when *ACBD3* was above the median level. ER+ patients had less OS when *ACBD3* expression was above the median (* *p* = < 0.05) (Figure 5f), but these changes were not as large as those seen in RFS differences (Figure 5c). ER+ patients had an upper quartile survival of 144 months when *ACBD3* expression was below the median and 105 months when *ACBD3* was expressed above the median level.

Distant metastasis-free survival (DMFS) was less likely when *ACBD3* expression was high: the upper quartile DMFS was 138 months for the cohort as a whole when *ACBD3* was expressed below the median level in breast tumor and 68 months when *ACBD3* was expressed above the median (Figure 5f). HER2− patients were at greater risk of distant metastasis when *ACBD3* was high in their tumors (Figure 5g). ER+ patients were more likely to have distant metastasis if tumor *ACBD3* was above the median level: upper quartile DMFS was 75 months when *ACBD3* was high and 143 months when *ACBD3* was low (Figure 5h). These findings in breast cancer patients indicate that high *ACBD3* mRNA expression negatively effects survival, relapse risk, and distant metastasis risk.

### 2.6. ACBD3 Expression in Responders and Non-Responders to Chemotherapy in Breast Cancer

**Figure 6:**
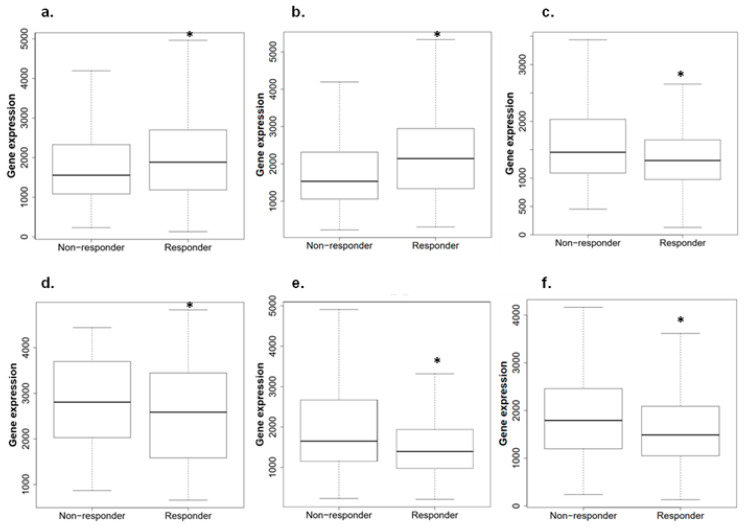
Breast tumor *ACBD3* expression in patients when divided by response or non-response to therapies. (**a**) *ACBD3* expression in breast chemotherapy responders and non-responders; *ACBD3* is 1.1 times higher in responders than in non-responders to chemotherapy overall in breast cancer (* *p* = 7.8 × 10^−6^). (**b**) *ACBD3* expression in HER2− chemotherapy responders and non-responders; *ACBD3* mRNA expression is 1.2 times higher in HER2− responders to chemotherapy (* *p* = 5.1 × 10^−11^). (**c**) *ACBD3* expression in HER2+ responders and non-responders to any chemotherapy; *ACBD3* is 1.2 times higher in HER2+ non-responders to chemotherapy (* *p* = 0.0029). (**d**) *ACBD3* expression in responders and non-responders to trastuzumab; *ACBD3* expression is 1.2 times higher in non-responders to trastuzumab (* *p* = 0.01). (**e**) *ACBD3* expression in responders and non-responders to lapatinib; *ACBD3* is 1.2 times higher in non-responders to lapatinib (* *p* = 0.011). (**f**) *ACBD3* expression in HER2+ responders and non-responders to anthracycline; *ACBD3* is 1.2 times higher in HER2+ non-responders to anthracycline (* *p* = 0.0023).

ROC plotter was queried to determine whether *ACBD3* expression had an impact on therapeutic outcomes [64]. *ACBD3* expression was higher in patients who had pathological complete response to combination chemotherapy regimens (FAC: 5-fluorouracil, doxorubicin, cyclophosphamide; FEC: 5-fluorouracil, epirubicin, cyclophosphamide; CMF: cyclophosphamide, methotrexate, 5-fluorouracil), as well as to individual agents such as ixabepilone, taxane, and anthracycline (Figure 6a). This observation is a strong contrast to data in Figure 5, where high ACBD3 expression had consistently negative patient outcomes. *ACBD3* expression was not significantly different between those that had 5 years relapse-free survival and those that relapsed before 5 years following chemotherapy (Figure 6b).

HER2+ breast cancer patients who had complete pathological response to chemotherapy had lower *ACBD3* RNA expression than those who did not respond (Figure 6c). Patients who responded to trastuzumab and lapatinib anti-HER2 therapies specifically also had significantly lower *ACBD3* expression than those who did not respond (Figure 6d,e). Anthracycline treatment also appeared to be more effective in patients with low *ACBD3* expression (Figure 6f).

### 2.7. Aberrant Protein Expression of ACBD3 in Breast Cancer Cores

**Figure 7:**
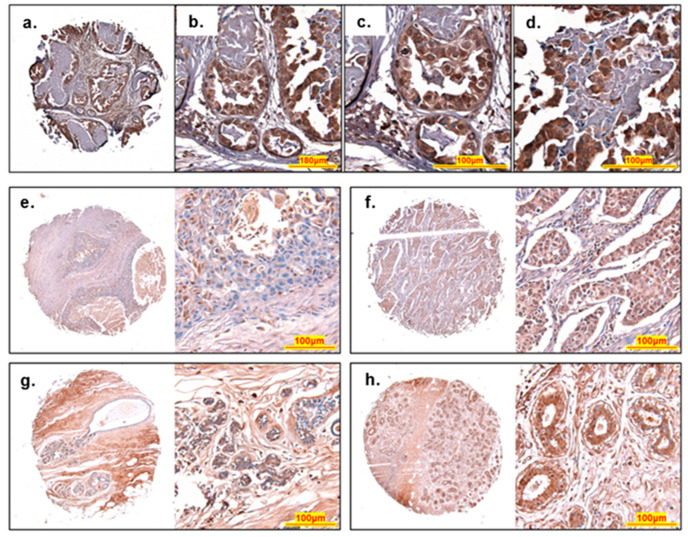
Histology of ACBD3 stained breast cores. (**a**–**d**) An invasive carcinoma from a 39-year-old female, stage IIA, ER- PR- HER2 2+ reveals a pattern of differential ACBD3 staining (brown), haematoxylin was used a as a nuclear stain (blue). (**a**) Low magnification image of entire core, fibrous interlobular tissue has a low level of ACBD3 staining whilst breast duct acini have high levels of ACBD3 staining. (**b**) Medium magnification of regular and irregular duct acini. (**c**) High magnification of regular small acini, shows high ACBD3 staining of luminal epithelial and basal myoepithelial cells. (**d**) High magnification of irregular large acini. Luminal epithelial and basal myoepithelial cells are both highly stained for ACBD3 but cells within the acini, possibly a ductal carcinoma in situ have a moderate-to-low level of ACBD3 staining, with some embedded cells with high levels of ACBD3, possibly luminal epithelial cells. (**e–h**) Typical ACBD3 staining of different cores. (**a**) Invasive ductal carcinoma of a 47-year-old female. Stage IA, grade 3, T1N0M0 scored as 1.1 overall for ACBD3 staining. (**b**) Invasive ductal carcinoma of a 50-year-old female. Stage IIA, Grade 2, T2N0M0 scored as a 2 overall for ACBD3 staining. (**c**) Normal-adjacent breast tissue with ductal ectasia of a 41-year-old female, scored 2.9 overall. (**d**) Cancer-adjacent tissue (adenosis) of a 39-year-old female, scored 1.7 overall.

Samples of human breast tissue from cancer patients were analyzed for ACBD3 expression by immunohistochemical staining. ACBD3 protein level expression in breast cancer is currently limited to 11 samples in the GEPIA database and a dataset from a single publication [22]. Biomax arrays with over 300 tissues cores were chosen to compare cancerous tissue with normal adjacent tissue, in addition to a comparison of expression between different breast cancer subgroups, with an emphasis on ACBD3 expression in receptor-positive versus receptor-negative breast cancers. Figure 7 represents typical ACBD3 staining of breast cancer tissue. There were areas of higher staining at the ducts or lobules with surrounding tissue showing low or no staining for ACBD3 (Figure 7a). At 40× magnification, individual cells of both regular and irregular lobules could be seen; luminal epithelial cells that line of the lobules had strong staining for ACBD3, as do the myoepithelial basal layer of cells beneath (Figure 7b). At 60× magnification, individual cells of the regular acini (Figure 7c) and irregular acini with invasive cells (Figure 7d) could be seen. Fibrous surrounding tissue had low staining for ACBD3, whilst the invasive cells had a strong mosaic pattern of high ACBD3 stained cells embedded within cells with low or no ACBD3 staining.

There were not many clear differences in ACBD3 staining based on patient pathology. The 1.55 mm cores of array BC08032a stained in a consistent and structure specific manner for cancer-adjacent tissue, normal-adjacent tissue, and breast cancer tissue. Figure 7e shows a core from an invasive ductal carcinoma, ACBD3 protein staining (brown) overall was low despite high coverage of haematoxylin nucleus staining (blue). At higher magnification of the central duct, ACBD3 expression fluctuated between cells. Fibrous tissue had moderate ACBD3 staining (bottom of the high magnification image (pink hue)). Figure 7f also shows an invasive ductal carcinoma and contained many more small ducts, and in this case, invasive cells in the ducts had higher staining of ACBD3 compared to the surrounding tissue. Invasive cells were tightly packed and showed a uniform amount of ACBD3 staining. Fibrous tissue appears to have low ACBD3 staining overall. Figure 7g shows a normal adjacent breast tissue core from a 41-year-old female with ducal ectasia. Adipose cells were visible at the lower right of the core and ACBD3 staining could not be seen here, in sharp contrast to the surrounding fibrous tissue where ACBD3 staining was higher. Increased magnification of the small ductal acini showed that staining was low in the benign ectasia cells blocking the ducts with some staining of ACBD3 at the basal myoepithelial cells. Figure 7h shows a core of cancer adjacent tissue with benign adenosis (enlarged numerous lobules). Epithelial and myoepithelial cells lining the ducts had high levels of ACBD3 staining with moderate staining of the surrounding tissue.

#### 2.7.1. ACBD3 Protein Expression in Malignant Breast Tissue and Metastatic Lymph Node Tissue

**Figure 8:**
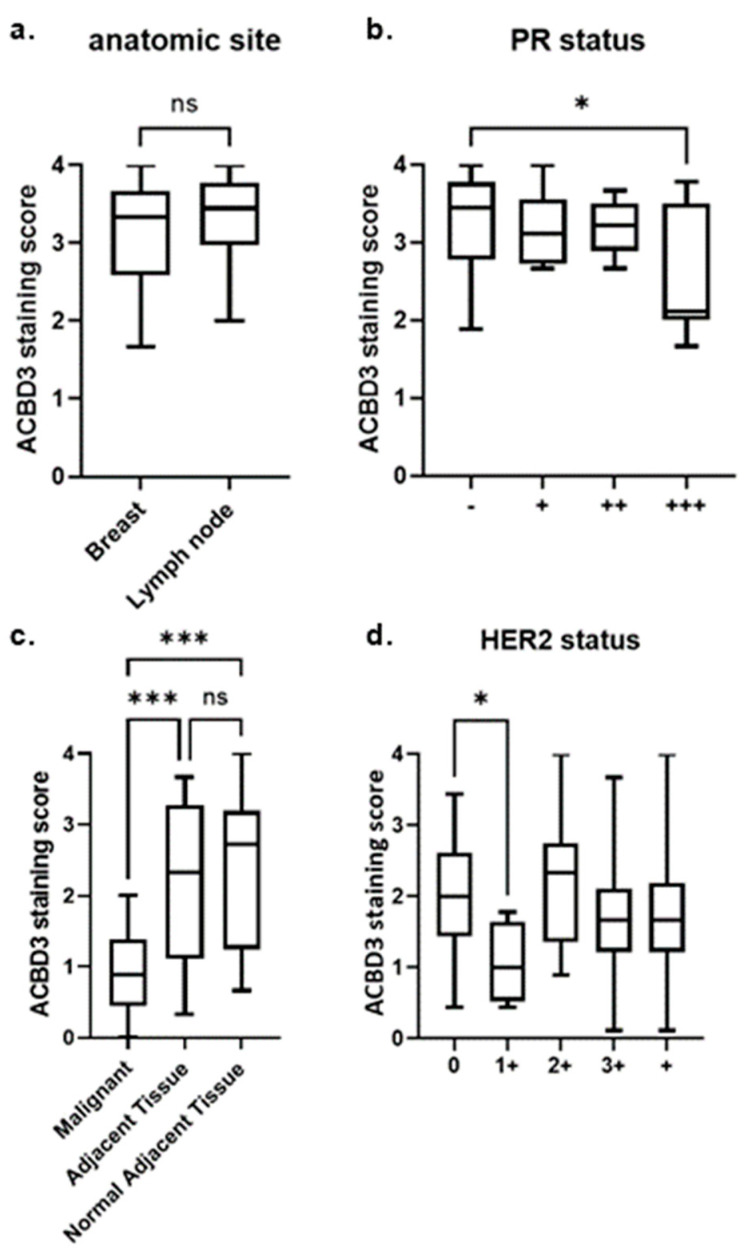
ACBD3 staining score of US BIOMAX fixed breast tissue core arrays. (**a**) Array BR1008B, there was no statistical difference in ACBD3 staining between malignant breast tissue (*n* = 48) and metastatic breast cancer of the lymph node (*n* = 38). (**b**) Array BR1008B, ACBD3 protein expression was significantly higher in PR negative breast cancer cores (*n* = 50) compared to PR 3+ cores (*n* = 9) (malignant breast tissue and metastatic lymph tissue, * *p* = 0.02202), but there was no statistical difference between PR− samples compared to all grades of PR+ core (not shown). (**c**) Array BC08032a, ACBD3 protein levels are significantly lower in malignant tissue compared to either cancer-adjacent tissue or normal-adjacent tissue, *** *p* < 0.001; ns = not significant (**d**) Array BR1401, there was a statistically significant difference between HER2− (grade 0) breast cancer samples and HER 1+ samples, * *p* = 0.0107. Error bars represent the standard deviation.

There was no statistical difference between ACBD3 protein expression between malignant breast tissue and metastatic breast cancer in lymph node (Figure 8a). High ACBD3 expression has previously been associated with more advanced-stage tumors and with cancer stem cells, so it was unexpected to find no statistical difference between non-metastatic and metastatic breast tissue (22). ACBD3 protein levels were significantly higher in PR− breast cancers (mean = 3.296) compared to breast cancers with high expression of PR (PR 3+) (mean = 2.642) (* *p* = 0.022) (Figure 8b). This included both non-metastatic and metastatic (lymph node) breast cancer samples, and this trend did not extend to PR− breast cancer samples when compared to samples with lower PR expression (PR 1+, PR 2+). Although most normal-adjacent tissue cores detached during staining, three partial cores were scored and had a mean score of 2.70; whilst this small sample size could not reach any statistical conclusions, it was lower than the mean average for the breast cancer tissue cores overall (3.25), which is in keeping with the previous literature but contradictory to the results of Figure 8, where ACBD3 protein expression was higher in breast cancer cell lines than a normal-like breast cell control [22].

#### 2.7.2. ACBD3 Protein Expression Is Lower in Malignant Breast Tissue Compared to Cancer Adjacent and Normal Adjacent Breast Tissue

Contrary to previous reports [22], ACBD3 protein staining results for this array found ACBD3 protein staining to be statistically lower in malignant breast cancer tissue compared to adjacent tissue or normal adjacent tissue (Figure 8c). The mean average ACBD3 staining for malignant tissue was 0.92/4 compared to 2.14/4 for adjacent tissue (*p* < 0.001), and 2.31 for normal adjacent tissue (*p* < 0.001). There was no significant difference in staining between the cancer-adjacent and normal-adjacent breast tissue samples. Comparing breast core staining by receptor status, subtype, grade, stage, or TNM score did not produce any differences that reached statistical significance within this limited sample.

#### 2.7.3. ACBD3 Protein Expression in Malignant Breast Tissue of Multiple Subtype Receptor Status and Pathology

Array BR1401 held no cases of breast cancer with PR 3+ receptor status, as such, the results from Figure 8b could not be corroborated in a larger sample size. HER2− breast cancer samples had significantly higher ACBD3 protein expression than HER2 1+ breast cancer samples (2.038, *n* = 49, versus 1.055, *n* = 4, respective mean averages, *p* = 0.0107) (Figure 8d). There was no significant difference between HER2− (0) breast cancer samples and HER2+ samples of any grade or between HER2 1+ and HER2 2+ breast cancer samples. The BR1008b array had no breast cancer samples with HER2 1+ staining, so this result could also not be corroborated between arrays.

No other statistically significant changes were found between subgroups of the sample by age, TNM score, grade, stage, pathology, or other receptor status (data not shown).

## 3. Discussion

The aim of this study was to determine whether ACBD3 is overexpressed in breast cancer, to investigate whether its expression impacts patient survival, and to consider the broader implications of breast cancer ACBD3 expression in terms of its interactions. We showed that *ACBD3* expression was almost doubled in breast cancer compared to healthy tissue but that changes in expression were not related to increased copy number or changes in methylation patterns, suggesting that other mechanisms were responsible for *ACBD3* over-expression. Several transcription regulators and regulatory pathways were associated with increased *ACBD3* expression in breast tissue, and some of these were linked to breast cancer. There was significantly less survival, more relapse, and more distant metastasis in ER+ patients when divided by *ACBD3* expression, and *ACBD3* overexpression appears to be universally detrimental to breast cancer prognosis.

IHC staining of ACBD3 in breast cancer patient core samples did not support previously published data [22]. A common feature of normal and cancerous tissue was high ACBD3 staining of luminal epithelial and myoepithelial basal cells of ducts. ACBD3 expression was found to be lower in cancer samples compared to adjacent tissue and normal adjacent tissue, which was not expected. Given that ACBD3 has previously been reported to be upregulated in breast cancer and that our bioinformatic data supported this, coupled with our survival analyses supporting previous findings [22], we do not believe that genetic heterogeneity of different cohorts can explain these differences. The most likely explanation is the difference in samples used. In our study, we compared tumor tissues with normal and normal-adjacent tissue from the same individuals. It is likely that the adjacent tissue is not a true representation of healthy tissue. Tissue samples adjacent to cancer have been found to possess qualities that are intermediary between normal and cancerous tissue [65]. Previously published work by Huang and colleagues [22] compared ACBD3 levels in tumor samples with normal tissue obtained from reduction mammoplasty, which could account for the differences observed in the two studies. As such, ACBD3 expression in normal-adjacent tissues may have been indicative of expression in intermediary tissue, and that is not truly normal. We hypothesis that ACBD3 levels may rise in normal tissues as part of the tumor development process, rendering high ACBD3 expression a potential marker of early-stage cancer or precancerous breast tissue.

High ACBD3 expression in breast cancer samples was associated with PR negativity and HER2 negativity. Nuclear staining of ACBD3 was clearly visible in examples in Figure 7 and supports findings of nuclear protein interactors SRSF2, a spliceosome and RNA export protein, and KDM2B, a histone lysine demethylase (supplementary Figure S1) and a previously reported ACBD3 nuclear localisation signal [1]. It is possible that this interaction occurs during mitosis when the nuclear envelope breaks down in the same way as src and Sam68 [66,67,68,69]. KDM2B inhibits senescence and, like ACBD3, is implicated in CSC self-renewal and Wnt/β-Catenin signaling in breast cancer [22,70,71]. Discovering that these proteins physically interact merits further study.

Invasive ductal carcinomas were observed to have many invasive cells in the lobules that had low staining for ACBD3, but some cells embedded in them had very high staining. As ACBD3 expression is associated with cancer stem cell formation, it is possible that this staining represents the formation of CSCs or cells that will become them. The increased risk of and decreased time to relapse and metastasis support a role for *ACBD3* in cancer stem cell formation and maintenance. By extension, an increased risk of metastases decreases survival, as 90% of cancer deaths are caused by metastatic tumors [72].

This study has found further evidence implicating ACBD3 in the Wnt signaling pathway and an additional ACBD3 association with CSC-maintaining proteins in breast cancer. This could explain why breast cancer patient relapse is more likely when *ACBD3* expression is high. It was unexpected to find that ACBD3 scoring was much lower for the cancers compared to either adjacent tissue, as this does not match the findings in cell lines, bioinformatics, or previous publications [22]. The fibrous and connective tissue in the adjacent tissues had more ACBD3 staining, and as the bulk of many cores were made up of this, they were highly stained. Fibrous tissue of the malignant cores had low-to-moderate staining with invasive cells that themselves had varying and sometimes strong staining for ACBD3. It is possible that the level of ACBD3 protein is negatively affected by tissue disruption. Another possible explanation is that ACBD3 protein expression was high in cancerous cells (as evidenced by high staining of irregular ductal epithelial cells and invasive cells of the duct), but that in the context of a whole core, including normal tissue and adipose tissue, ACBD3 was not increased. ACBD3 may even be the target of downregulation in response to breast cancer that cancerous cells do not respond to but that normal cells surrounding them do. There is some precedent for this, as ACBD3 was found to be suppressed by ER signaling but is highest in ER+ cell lines, suggesting that repressors of ACBD3 may be reprogrammed in breast cancer. ACBD3 staining was significantly higher in PR- breast cancer cores compared to PR 3+ cores on one array and significantly higher in HER2− cores compared to HER2 1+ cores on another array, but this result could not be cross-correlated between slides because only one slide had PR 3+ breast cancer patients and that same slide held no HER2 1+ patients.

High ACBD3 expression was associated with poorer distant metastasis-free survival in ER+ breast cancer patients. ER pathway related GNST and 17βE2 negatively regulated ACBD3 in breast tissue. FOXA1 is a forkhead DNA binding protein transcription factor that is essential for oestrogen receptor α expression and is involved in breast morphogenesis [73]. FOXA1 has been found to be commonly expressed in breast metastases, coordinated with ER expression in these metastases and mediated ER-binding reprogramming [74,75]. It is conceivable that ER signaling may regulate ACBD3 in breast cancer expression, but that FOXA1 contributes to the reprogramming of ER binding and signaling in breast cancer [74], cancelling out the negative impact of GNST and 17βE2-mediated ER transcriptional repression, explaining why decreased distant metastasis-free survival was observed in ER+ patients when *ACBD3* expression was high. High *ACBD3* expression in ER+ patients was associated with poorer relapse-free survival (Figure 5c) supporting an interplay between ER signaling and ACBD3. The xenoestrogen bisphenol A (BPA), an endocrine disrupter, was also found to increase ACBD3 transcription in breast tissue [76,77]. BPA exposure has previously been linked to breast cancer [78,79].

*ACBD3* levels were significantly higher in non-responders to anti-HER2 therapy and *ACBD3* transcription was induced by X10, an insulin analogue that activates the IGF and insulin receptors. The insulin and IGF1 receptors have important roles in breast cancer; IGF signaling is important in mammary gland development and metastatic pathways, and its receptor is overexpressed and hyperphosphorylated in breast cancer. The insulin receptor is now being explored as a target for therapy [12,25,80,81,82]. IGFIR can phosphorylate and activate the HER2 receptor to negate the effects of anti HER2 therapies in breast cancer cell lines and anti-IGFIR drugs re-sensitize trastuzumab-resistant cell lines to trastuzumab [83,84,85,86]. Although an artificial and potent insulin analogue, X10 strongly upregulated *ACBD3* transcription in healthy breast tissue. X10 is reported to cause breast cancer in Sprague–Dawley rats and had a mitogenic effect in MCF7 cells [87,88,89]. X10 has also been seen to increase the growth of MC38 colon cancer allografts on obese mice and increased mammary tumor occurrence in rats [90]. It is conceivable that IGF1R signaling induces ACBD3 expression, which would increase the pool of available GLUT4-containing vesicles and therefore increase glucose import and energy for the proliferating cancer cells, propagating the Warburg effect. Higher expression of *ACBD3* in non-responders to trastuzumab may be an indicator of the increased IGF signaling that sustains HER2 activation and signaling in the presence of trastuzumab.

## 4. Materials and Methods

### 4.1. Bioinformatic Analysis

Expression of ACBD3 was validated by the gene expression profile interactive analysis resource (GEPIA) (http://gepia.cancer-pku.cn/ accessed 22 June 2022) using the GTEx and TCGA databases [35].

Copy number variation and mutations were retrieved from the TCGA cohort pan-cancer data with the cBioPortal resource (http://www.cbioportal.org/ accessed 5 August 2022) [36,37]. Gene variants and upstream intergenic variants were retrieved from the Genome-Wide Association Studies Catalogue (https://www.ebi.ac.uk/gwas/ accessed 22 June 2022) and from Genehancer (https://www.genecards.org/ accessed 22 June 2022) [38,43]. Pan-cancer analysis data sampled: ACC, adrenocortical carcinoma; BLCA, bladder urothelial carcinoma; BRCA, breast invasive carcinoma; CESC, cervical squamous cell carcinoma and endocervical adenocarcinoma; CHOL, cholangiocarcinoma; COAD, colon adenocarcinoma; DLBC, lymphoid neoplasm diffuse large B cell lymphoma; ESCA, oesophageal carcinoma; GBM, glioblastoma multiforme; HNSC, head and neck squamous cell carcinoma; KICH, kidney chromophobe; KIRC, kidney renal clear cell carcinoma; KIRP, kidney renal papillary cell carcinoma; LAML, acute myeloid leukaemia; LGG, brain lower grade glioma; LIHC, liver hepatocellular carcinoma; LUAD, lung adenocarcinoma; LUSC, lung squamous cell carcinoma; MESO, mesothelioma; OV, ovarian serous cyst-adenocarcinoma; PAAD, pancreatic adenocarcinoma; PCPG, pheochromocytoma and paraganglioma; PRAD, prostate adenocarcinoma; READ, rectum adenocarcinoma; SARC, sarcoma; SKCM, skin cutaneous melanoma; STAD, stomach adenocarcinoma; TGCT, testicular germ cell tumors; THCA, thyroid carcinoma; THYM, thymoma; UCEC, uterine corpus endometrial carcinoma; UCS, uterine carcinosarcoma and UVM, uveal melanoma.

Methylation data were retrieved from TCGA and MET500 OMICs data using the UALCAN resource (http://ualcan.path.uab.edu/ accessed 22 June 2022) [44].

Breast cancer patient relapse-free survival, overall survival, and distant metastasis-free survival data were retrieved and analyzed into Kaplan–Meier survival curves in the KMplotter resource using the 202324_s-at ACBD3 dataset (https://kmplot.com/ accessed 22 June 2022) [63,91]. ACBD3 expression data in breast cancer patient responders and non-responders to therapies were retrieved from ROCplotter (http://www.rocplot.org/ accessed 22 June 2022) [64].

ACBD3 binding factors and transcription factors were found using the Signaling Pathways Project resource (https://www.signalingpathways.org/ accessed 5 August 2022) to probe manually curated ChIP-Seq and transcriptomic data [51].

ACBD3 protein data were retrieved from the Human Protein Atlas (https://www.proteinatlas.org/ accessed 222 June 2022) [92]. Association data for protein interactions and co-expression were carried out using geneMANIA (genemania.org accessed 22 June 2022) [93].

Factors that affected ACBD3 transcription and interactors of ACBD3 were assessed for their relevance to breast cancer by extensive literature searches. Pathways with strong established links to breast cancer such as those in the oestrogen or insulin pathway were prioritized.

### 4.2. Immunohistochemistry

Breast core tissue arrays (Biomax, Derwood, MD, USA) were stained using standard protocols. Briefly, paraffin was removed with histoclear and ethanol, heated with sodium citrate, washed in 0.025% triton-X PBS, then incubated in 3% hydrogen peroxide PBS for 5 min. The slides were then blocked with BSA, incubated with ACBD3 primary antibody (Abcam), then incubated with a biotin-labelled anti-rabbit secondary antibody and streptavidin-HRP with 0.025% triton-X PBS wash steps between each incubation. DAB solution (Zytochem) was added to visualize ACBD3 staining, and haematoxylin was used as a nuclei stain. Each core was scored per 1/3 core as 0–10% staining = 0, 10–25% staining = 1, 25–50% staining = 2, 50–75% staining = 3, 75–100% staining = 4; all cores were scored on three sequential days and the mean score for all days was taken. Staining was also independently scored and compared (Figure S2). The specificity of ACBD3 antibody was verified by use in western blots producing a band of expected size.

Array BR1008b consisted of 101 cores: 1 adrenal cortex control core, 50 cores of malignant non-metastatic breast cancer tissue of various stage, grade, and receptor status. Forty cores were of breast cancer metastasis into lymph node tissue, and ten cores were of normal adjacent tissue. Array BC08032a consisted of 64 cores: 1 adrenal cortex control core plus equal parts malignant breast tissue, cancer-adjacent breast tissue, and normal-adjacent breast tissue. Array B1401b consisted of 141 cores: 1 adrenal cortex control core and 140 cores of breast cancer tissue of various stage, grade, receptor status, and pathology.

### 4.3. Core Analysis

Scores for ACBD3 staining on independent days were analyzed for each array and presented as Bland Altman plots (Supplementary Figure S2) [94]. In all cases, the bias value (the average of the difference in score) was very small, indicating that scores were not biased between days and were therefore equally valid. Difference in ACBD3 staining score between days was smallest at high score value (4 out of 4) for all three arrays. The +/−95% limit of agreement and hence the difference was smaller between the data presented here and the data generated by an independent scorer than the difference between repeated measurements by our scorer on sequential days. This highlights the importance of taking a mean average of repeated measurements and also suggests that the techniques between our scorer and an experienced tissue core scorer were consistent. The bias was very low between our scorer and the other scorer, but our scores were consistently slightly higher across all arrays (Supplementary Figure S2c,f,i).

There was a clear trend in the BR18008b array, where difference in score between days was highest for scores of 3, decreasing for lower or higher scores (Supplementary Figure S2d,e). This suggests that scoring cores around 75% ACBD3 staining intensity was the least consistent and most susceptible to ambiguity. This array also had fewer low ACBD3 intensity scored cores (minimum mean score = 1.3).

To a lesser extent, there was also a trend in the BR1401b array towards larger differences between days around scores of 2, but there was a more even distribution of scores overall (Supplementary Figure S2g,i). Larger differences between days for scores between 2 and 3 may be down to the heterogeneity of samples and therefore ACBD3 staining, making it harder to consistently score cores with middling overall ACBD3 protein staining.

The relatively thick cores (5 micron) prevented automatic reading of the arrays by computer, as the reader could not focus on cores consistently. Overall, the scoring was not biased from day to day, but there was some ambiguity in scoring. The identity of the individual cores was not known until after all scoring was complete and scores from previous days were not observed when repeating measurements.

The interclass correlation coefficient (ICC) was also calculated for the data compared to Figure S2. First, the ICC was calculated using the two-way mixed effects model to measure consistency between the scorers (a research team scorer and an independent scorer model [95]. The ICC score between scorers for the BC08032a array slide was 0.923, meaning that reliability between scores was excellent. For the BC1008b array, the ICC was 0.741, indicating moderate reliability bordering good reliability. The ICC score for the BR1401 array equalled 0.733, very similar to the score for the BC1008b array.

The ICC between days of scoring was then calculated. As this was undertaken by a scorer on our research team, the test type was test/retest. The model was the same, but the ICC score dictates absolute agreement and not consistency. ICC between repeated measures of the BC08032a array was 0.913, indicating excellent agreement between repeated scoring. The ICC score for BC1008b was 0.699, indicating moderate agreement between days; as in the Bland–Altman plots (Figure S2d,e), there was clearly more discrepancy in score for this array than in others. The ICC value for the BR1401 scoring was 0.822, indicating that there was good agreement between scores on different days.

The data collected from the arrays was relatively small at 50 samples or less per condition queried and group sizes were uneven. Because of this, Shapiro–Wilk analysis was carried out to determine if the distribution of scoring for subgroups departed from normal distribution. Unpaired t-tests were performed on data that appeared to be normally distributed. There was evidence that data from the BR1008b array were not normally distributed, so the Mann–Whitney unpaired non-parametric test was used to determine *p* values for these data.

Scoring of the BR1008b array was generally high and close to the natural limit of 4, causing a distribution skew to the left. Figure 8a looked broadly at local breast cancer tissue vs. breast cancer tissue that had invaded the lymph node; the data for both groups were skewed to the left (high stain scores) and had some resemblance of a bimodal distribution. A bimodal distribution was not unexpected, as within the dataset were patients of different ages, tumor receptor statuses, tumor grades, and stages. Stratifying by one or more of these variables may lead to binomial distribution of the data but also would reduce the size of the dataset significantly.

Rather than stratify the anatomic datasets, patients were instead divided by PR status (Figure 8b). PR− cores did not follow a normal binomial distribution but did have a a high frequency of scoring close to the natural limit (W = 0.9221, * p = 0.002807, *n* = 50). As before, PR− patients also had several other variables such as ER status, age, and tumor grade, which could have caused the bimodal distribution of data seen. The PR3+ cores did not show evidence of non-normality (W = 0.8506, * p = 0.08112, *n* = 9). The Mann–Whitney test was used to compare these data (* p = 0.02202)

Array BC08032a (Figure 8c) contained fewer cores and was analyzed for ACBD3 expression across malignant, adjacent, and normal-adjacent tissue. There was no evidence for non-normality of adjacent tissue core scoring (W = 0.9196, * p = 0.085, *n* = 21). There was no evidence for non-normality of malignant core scoring (W = 0.9585, * p = 0.487, *n* = 21). The normal-adjacent tissue score distribution departed significantly from normality (W = 0.8992, * p = 0.0398, *n* = 20); these data appeared to be skewed to the left and when Box–Cox power transformation was performed with a λ of 1.5, there was no longer evidence for non-normality (W = 0.9216, 0.09325), suggesting skew towards the natural limit rather than non-normality.

HER2 0 cores on array BR1401 (Figure 8d) showed no evidence of non-normality (W = 0.9827, * p = 0.9997, *n* = 4). HER2 1+ cores on array BR1401 showed no evidence of non-normality (W = 0.9705, * p = 0.2529, *n* = 49).

## 5. Conclusions

The location of ACBD3 on chromosome 1q and recent findings of a CSC promoting role in breast cancer make ACBD3 a candidate for further study in breast cancer research. Little is known about the regulation of ACBD3, despite the number of important cellular pathways it participates in, and there is a wide scope for a breast cancer role beyond CSC formation by Wnt signaling. Due to the breadth of possibilities, it was pertinent to first analyze bioinformatic resources to narrow the field of study and to form hypotheses for in vitro and ex vivo work. The results presented are almost exclusively correlative and require further study to verify.

ACBD3 appears to be a candidate biomarker for poor patient prognosis in breast cancer and may possibly be a biomarker for ER signal reprogramming or of precancerous breast tissue. The findings here also support the current evidence for ACBD3 involvement in breast cancer stem cells.

Relationships between ACBD3 and all three major breast cancer hormone and signaling receptor pathways (ER, HER2, and PR) have been found. High ACBD3 expression was found to be detrimental to breast cancer patient outcomes across many subgroups, and there were also small changes in response to certain therapies depending on ACBD3 expression. Interaction with one of the major breast cancer hormone receptor pathways could explain these results, indicating that there is a real need for study into the ACBD3-(ER, PR, HER2) relationships. Such knowledge will shed light on the potential of ACBD3 as a possible therapeutic target and/or prognostic biomarker.

## Data Availability

No datasets were generated during this study.

## Supplementary Materials

The following supporting information can be downloaded at: https://www.mdpi.com/article/10.3390/ijms23168881/s1.
