# Supplementary figures and images for: ACBD3 Bioinformatic Analysis and Protein Expression in Breast Cancer Cells
Source: Int J Mol Sci. 2022 Aug 10;23(16):8881. doi: 10.3390/ijms23168881 (PMC9408326; doi:10.3390/ijms23168881)

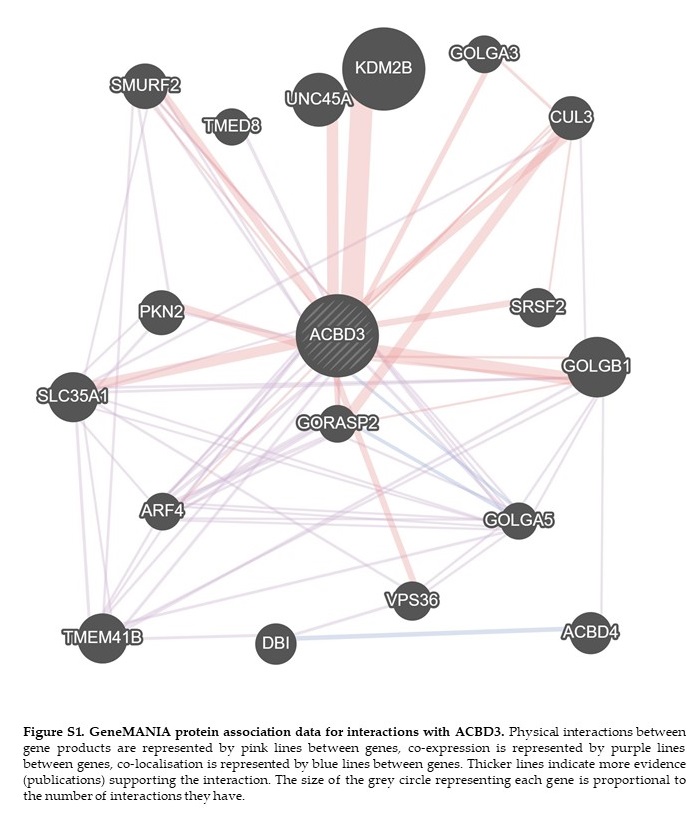

Supplement: Supplementary file 1 [file ijms-23-08881-s001.zip › Figure S1.jpg]

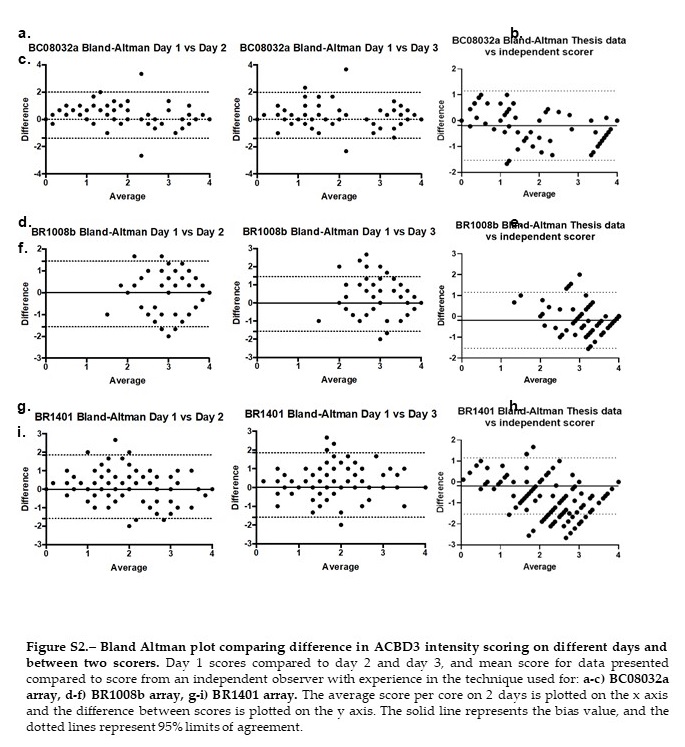

Supplement: Supplementary file 1 [file ijms-23-08881-s001.zip › Figure S2.jpg]
